# Defining Multiple Characteristic Raman Bands of α-Amino Acids as Biomarkers for Planetary Missions Using a Statistical Method

**DOI:** 10.1007/s11084-015-9477-7

**Published:** 2016-01-07

**Authors:** S. M. Rolfe, M. R. Patel, I. Gilmour, K. Olsson-Francis, T. J. Ringrose

**Affiliations:** Planetary and Space Sciences, Department of Physical Sciences, The Open University, Robert Hooke Building, Walton Hall, Milton Keynes, MK7 6AA UK; Space Science and Technology Department, STFC Rutherford Appleton Laboratory, Chilton, Didcot, Oxfordshire, OX11 0QX UK; Department of Environment, Earth and Ecosystems, The Open University, Milton Keynes, MK7 6AA UK

**Keywords:** Raman spectroscopy, Amino acids, Biomarker, Mars, Astrobiology, ExoMars

## Abstract

Biomarker molecules, such as amino acids, are key to discovering whether life exists elsewhere in the Solar System. Raman spectroscopy, a technique capable of detecting biomarkers, will be on board future planetary missions including the ExoMars rover. Generally, the position of the strongest band in the spectra of amino acids is reported as the identifying band. However, for an unknown sample, it is desirable to define multiple characteristic bands for molecules to avoid any ambiguous identification. To date, there has been no definition of multiple characteristic bands for amino acids of interest to astrobiology. This study examined l-alanine, l-aspartic acid, l-cysteine, l-glutamine and glycine and defined several Raman bands per molecule for reference as characteristic identifiers. Per amino acid, 240 spectra were recorded and compared using established statistical tests including ANOVA. The number of characteristic bands defined were 10, 12, 12, 14 and 19 for l-alanine (strongest intensity band: 832 cm^-1^), l-aspartic acid (938 cm^-1^), l-cysteine (679 cm^-1^), l-glutamine (1090 cm^−1^) and glycine (875 cm^-1^), respectively. The intensity of bands differed by up to six times when several points on the crystal sample were rotated through 360 °; to reduce this effect when defining characteristic bands for other molecules, we find that spectra should be recorded at a statistically significant number of points per sample to remove the effect of sample rotation. It is crucial that sets of characteristic Raman bands are defined for biomarkers that are targets for future planetary missions to ensure a positive identification can be made.

## Introduction

Amino acids, among several molecules, have been identified as potential candidates for detection in the search for life elsewhere in the Solar System (e.g., Parnell et al. 2007; Aerts et al. 2014). Extraterrestrial amino acids have already been detected in meteorites, for example, the Murchison meteorite (e.g., Kvenvolden et al. 1970) and the Orgueil and Ivuna meteorites (Ehrenfreund et al. 2001). Murchison was found to harbour amino acids that are unique to carbonaceous chondrites, with only eight of the 75 detected amino acids present in terrestrial biology (e.g., Kvenvolden et al. 1970; 1971; Cronin et al. 1995). As these studies show, the detection of amino acids is not an unambiguous indicator of life, as there are many amino acids not used by life and a full catalogue of the amino acids that are have not been discovered elsewhere in the Solar System to date. Nonetheless, amino acids are regarded as a high priority for detection (Parnell et al. 2007) due to their ubiquity in terrestrial life as the building blocks of biologically important biomacromolecules, such as proteins. Although other biomarkers could be considered as unambiguous indicators of life, such as porphyrin, which is only produced in large quantities by biological processes (Suo et al. 2007) and β-carotene (Vítek et al. 2009), detecting a localised concentration of all 21 amino acids exclusively used by terrestrial biology in an extraterrestrial environment such as Mars could suggest that life similar to that found on Earth may have arisen there in the past.

The Curiosity rover is searching for organic molecules at the surface of Mars (Ming et al. 2014; Webster et al. 2014; Stern et al. 2015); if organic molecules formed on Mars, current surface conditions are not conducive to their survival and they may be difficult to detect. Highly reactive salts, such as perchlorates as discovered by the Phoenix lander (e.g., Hecht et al. 2009) may have contributed to ambiguity over the interpretation of the life detection experiments from the Viking missions; it has been suggested that organics were present in Viking regolith samples, but their detection was inhibited by the presence of perchlorate (e.g., Levin and Straat 1979; Navarro-González et al. 2010). Nevertheless, previous studies have constrained the potential survivability of amino acids on the surface of Mars. For example, Johnson et al. (2011) found that the half-life of l-alanine, l-valine, l-aspartic acid, l-glutamic acid and glycine exposed to simulated martian conditions was 132 ± 87 days with exposure to ultraviolet radiation (UV) and 477 ± 295 days without exposure to UV. In a more detailed study by ten Kate et al. (2005), thin films of glycine and d-alanine were exposed to UV radiation to calculate the rate of photodestruction. They found that a concentration of 1 ppb of amino acids in the martian regolith would degrade to a concentration of 1 ppt in 5 × 10^6^ years for D-alanine and 4 × 10^7^ years for glycine, indicating that amino acids, which may have arisen on early Mars, could still be present on the surface today at sub-ppt levels.

On future planetary missions, if amino acids are detected they will need to be distinguished from a matrix of materials, such as regolith. The detection of biomarkers, molecules that cannot be synthesised in large quantities by abiogenic means (Eglinton et al. 1964), is a priority for the ESA ExoMars mission in 2018. A Raman spectrometer equipped with a 532 nm laser (Edwards et al. 2012a) will be among the suite of instruments on board to aid the detection process (Edwards et al. 2012b). This excitation wavelength was chosen for the range of molecular targets that could be acquired, while reducing background fluorescence and being able to equip the rover with a commercially available laser that would meet the mission mass and power requirements (Edwards et al. 2012a). Raman spectroscopy has several potential advantages over other methods of life detection: it provides a spectroscopic “fingerprint” of molecules, samples require little or no preparation prior to analysis, samples are not destroyed by the analysis, and water does not interfere with the “fingerprint” region, which is between 500 and 1700 cm^-1^ (Auer and Skinner 2008). There have been several terrestrial studies that have attempted to assess the efficacy of Raman spectroscopy as a biomarker detection tool in extraterrestrial environments. For example, the Marsokhod test rover demonstrated that Raman spectroscopy could be used to identify carotenoids in samples identified by the rover team as biologically interesting (Newsom et al. 2001). An extensive study of extremophilic organisms using a range of Raman excitation wavelengths sought to determine optimal analytical parameters (Jorge Villar et al. 2005). An infrared (IR) laser at a higher excitation wavelength of 1064 nm required long exposure times to collected useful spectra, especially for compounds of low concentration and lower wavelengths such as 514 and 488 nm lasers were not able to obtain good results for geomarkers; they concluded that an excitation wavelength of 785 nm provided a compromise for the study of both biological and geological samples in extraterrestrial environments (*ibid*). The conclusion that the 785 nm wavelength laser provided a compromise appears well founded; however, not all laser wavelengths were used on all the samples and the concentrations studied are not defined, making it unclear the importance that should be placed on this part of the results. Furthermore, when studying halophilic cyanobacteria using a 514 nm wavelength laser, it was possible to identify the presence of two different species. Though good results were obtained from the 785 nm wavelength when searching for biomarkers such as carotene bands, the 514 nm wavelength seemed to have provided a greater depth of information by being able to distinguish between species. Other samples that were analysed often showed strong biomarker spectra with the 514 nm wavelength, therefore, microbial biomarkers were more accessible with the mid-optical range of wavelengths. Portable Raman instruments, using an excitation wavelength of 785 nm, were successfully used to determine the Raman spectra of solid amino acids that had been exposed to outdoor conditions: full sunshine and an ambient temperature of 20 °C (Culka et al. 2010). Similarly, Culka et al. (2011; 2012) examined several biomarker/matrix mixes using portable Raman devices and were able to identify the individual biomarkers, including glycine and alanine, from their Raman signatures within the gypsum matrix.

Qualitative analyses of the protein amino acids and other molecules have been carried out with Raman spectroscopy (e.g., Jenkins et al. 2005; De Gelder et al. 2007; Zhu et al. 2011). These studies present the spectra for reference and have largely described the strongest Raman bands as those that might best serve as identifiers for specific molecules. For glycine and alanine, intense bands were found in the region 850 to 900 cm^-1^, assigned to the symmetric CNC stretch mode (De Gelder et al. 2007). For glycine, the strong bands at 894 and 1327 cm^-1^ were considered markers; the marker for alanine is reported as the strong band at 852 cm^-1^; and for aspartic acid, the band at 939 cm^-1^ is described as the most intense, with another strong intensity band at 1338 cm^-1^, by Zhu et al. (2011). Furthermore, Rosado et al. (1997) and Kumar et al. (2006) described the bond assignments for the bands of alanine; bond assignments for aspartic acid, cysteine and glycine are reported by Navarrete et al. (1994), Pawlukojć et al. (2005) and Kumar et al. (2005), respectively. Bond assignments for glutamine are defined by Dollish et al. (1974) and Dhamelincourt and Ramirez (1993).

However, identification of amino acids based on these studies is complicated by several factors. 1) Identification of characteristic spectral bands for individual amino acids have been based on the position of the strongest band and have not considered weaker bands that may prove useful in identifying amino acids in complex matrix materials. Although studies such as Culka et al. (2011; 2012) follow good practise and use multiple bands as reported elsewhere (e.g., Kumar et al. (2005) for the Raman bands of l-alanine) to identify molecules within a matrix of materials, it has been noted previously by Vandenabeele et al. (2012) that it may be necessary to define the minimal number of bands needed to make a positive identification of a molecule when in a mixed material. 2) Studies that have published relative wavenumber positions of amino acid bands have often examined the samples with single but differing excitation wavelengths and Raman systems, which has led to discrepancies in intensity of bands and of up to 33 cm^−1^ when comparing results between studies (e.g., Kumar et al. 2006; Zhu et al. 2011). Furthermore, a range of excitation wavelengths will be used on future planetary missions (e.g., 532 nm on ExoMars; 248.6 nm on Mars 2020, which is highly sensitive to condensed carbon, aromatic organics and minerals relevant to aqueous chemistry (Beegle et al. 2014)), so it is important to study the same astrobiologically relevant molecules with various excitation wavelengths.

Following these points, in this study a quantitative statistical approach was adopted to define the characteristic bands for l-alanine, l-aspartic acid, l-cysteine, l-glutamine and glycine. Multiple characteristic bands are presented as reference for each of the Raman bands of these astrobiologically relevant molecules. Furthermore, band intensities are provided and reference spectra of the amino acids are displayed for future studies of these amino acids.

## Methods

### Experimental Method

Samples of l-alanine, l-aspartic acid, l-cysteine, l-glutamine (hereafter referred to as alanine, aspartic acid, cysteine, and glutamine) and glycine (99+ % purity; Sigma Aldrich, UK) were prepared as 1 % solutions in 0.1 M HCl. Each amino acid, in triplicate, was pipetted onto individual glass Petri dishes and dried at 65 °C overnight. This resulted in the formation of a thin film of solid amino acid approximately between 4 and 10 mm in diameter. The samples were examined using a HORIBA Jobin Yvon ‘LabRAM HR’ bench top Raman spectrometer, equipped with 514 and 473 nm lasers and a charge-coupled device (CCD), which was cooled to −70 °C. The grating had 600 grooves per mm and the objective lens (×50) provided a spot size of 0.8 μm. The power of each laser was 4.56 ± 0.27 mW and 4.49 ± 0.27 mW for the 514 and 473 nm excitation lasers, respectively. The accuracy in the measurement of the wavenumber position of the bands is ±0.98 cm^-1^, defined by the resolution of the grating, where measurements of the intensity occur on average every 1.96 cm^-1^.

### Calibration and Data Collection

To ensure the hardware and software were in the correct configuration after switching between excitation lasers, prior to data collection the Raman spectrometer was adjusted to a zero position value of 0.0 cm^-1^ using white light. To calibrate the band position of the Raman spectra using the laser light source (either the 514 or 473 nm laser; the same source that was used to examine the amino acid samples) using a chip of pure silicon as a standard. The spectrometer coefficient was adjusted until the silicon band was at the accepted calibrated value of 520.7 cm^-1^. The calibration ensured that the instrument was in an identical initial state for each sample.

For each amino acid, three maps (100 × 100 μm), consisting of 16 spectra in a 4 × 4 grid with equal spacing, were produced for the 514 and 473 nm laser excitation wavelengths. Each map position was imaged and co-ordinates from a designated origin point were noted. This allowed the three maps to be measured in both wavelengths for direct comparison. The triplicate maps will be referred to as map 1, map 2 and map 3. The integration time was 5 × 15 s exposures, a total integration time of 75 s, for each spectrum. To avoid the Rayleigh scattering band overwhelming the less intense Raman signature, the Raman shift with a range of 10 to 2000 cm^-1^ was examined, which incorporates the “fingerprint” region, 500 to 1700 cm^-1^ (Auer and Skinner 2008). Each spectrum was processed using the software *LabSpec5* to correct for effects due to the background signal, which is comprised of fluorescence effects, noise and offset of the CCD detector and other optical components of the spectrometer and stray ambient light.

The first part of the process in determining whether the bands in each spectrum were characteristic of the amino acid was to check the frequency of occurrence of each band in the 48 spectra; the upper quartile of the sample bands were categorised as potential characteristic bands of that amino acid. These bands were subjected to further statistical analysis, described in The Definition of a Characteristic Band and Statistical Methods, to identify characteristic bands for each amino acid. With every sample there were bands that did not fall into the upper quartile, though these bands may well be associated with the particular amino acid, they may not have been sufficiently intense to be identified by the band fitting algorithm over the 16 points in the map. This could be due to the bond associated with the band having an innately weak Raman scattering effect or the scattered light was not received by the instrument due to the angle of the crystal surface. Furthermore, though measures were taken to ensure that the samples remained pure, this upper quartile categorisation allows for bands that may not be associated with the amino acids due to contamination to be ruled out, leaving only those that are statistically significant. Occasionally there was a band that is only in the upper quartile range in two of the three maps. This was repeated for each excitation wavelength.

### The Definition of a Characteristic Band and Statistical Methods

Statistical approaches have previously been effectively used to characterise Raman spectra of polymorphs of pharmaceutical compounds and therefore distinguish them based on the wavenumber position of the bands (e.g., Mehrens et al. 2005). The aim of applying statistical tests to the Raman bands of the amino acids in this study was to define a set of characteristic bands that can be reliably used to identify the molecules in samples such as the martian regolith. The spectra from the triplicate maps of each amino acid were tested against one another using the Analysis of Variance (ANOVA) and Tukey-Kramer statistical tests (described below) to check the consistency of the band so a set of characteristic bands could be selected.

Bands were defined as characteristic bands of the amino acid molecule if they satisfied the following criteria: 1) the band appeared in all three maps and 2) the band had an ANOVA test *p* value of ≥ 0.05. If a band did not pass the ANOVA test, but either of the following criteria were met: 3) the intensity of the band is classified as very strong and/or 4) the means of the triplicate maps were within the instrument measurement accuracy of ± 0.98 cm^-1^, the band was classified as characteristic.

Tentative bands that could be used to aid identification of the molecule are also considered, for example, a band may have been close to passing the ANOVA *p* value test but, the band position in one of the maps had a greater difference in relative wavenumber than the other two band positions, causing the *p* value to be <0.05. Therefore the wavenumber position of these bands is not as consistent as the characteristic bands. By satisfying at least one of the following criteria: T1) pass one or more of the post-hoc Tukey-Kramer tests; T2) the means of two out of the three maps are within the measurement accuracy; T3) have an intensity rating of medium-weak or above (band intensity classification is described below); T4) have a standard deviation of 1.00 or less. Tentative bands should not be considered as characteristic bands.

The intensity classification of the Raman bands in this investigation follow the classification criteria as defined by Vandenabeele et al. (2012), to provide consistency in the field of Raman spectroscopy. These annotations have been applied in this study: very strong (vs), strong (s), medium-strong (m-s), medium (m), medium-weak (m-w), weak (w) and very weak (vw).

Ambiguous bands, here defined as those that were recorded as singlets in some map points and doublets or shoulders in others, were not considered when selecting bands as characteristic of the amino acid for use as identifiers. Secondly, *t*-tests were applied to the spectra of the amino acids in both excitation wavelengths, to test the consistency of the band position. For this, the term relative wavenumber difference (RWD) is used to define the consistency and hence the presence of the bands in spectra, either within or between wavelengths. The RWD of the amino acid bands were compared in each triplicate map to aid in the identification of characteristic bands.

The null hypothesis assumed that the mean of the band positions were the same and therefore, no RWD had occurred. The ANOVA test was performed to test the difference between the means of the bands between the three maps. Used in combination with the ANOVA test, the Tukey-Kramer test compared all possible pairs of means, even if there was an uneven sample number. Both one- and two-tailed *t*-tests were used to compare the band positions between the two wavelengths as it was unknown in which direction (to higher or lower wavenumbers) the RWD would occur. The results from these tests were used to determine the consistency of the band position and therefore their usefulness as characteristic bands. For ANOVA, Tukey-Kramer and the *t*-tests there was an output of a *p* value, which denoted the probability between 0 and 1 that the measurements observed could have occurred by chance. A significance level (α) of 0.05 is used. The ANOVA test also outputs a value known as *F. F* is equal to 1 if there has been no effect as a result of treatment. *F* becomes increasingly larger the more inconsistent it becomes with the null hypothesis.

## Results

**Fig. 1:**
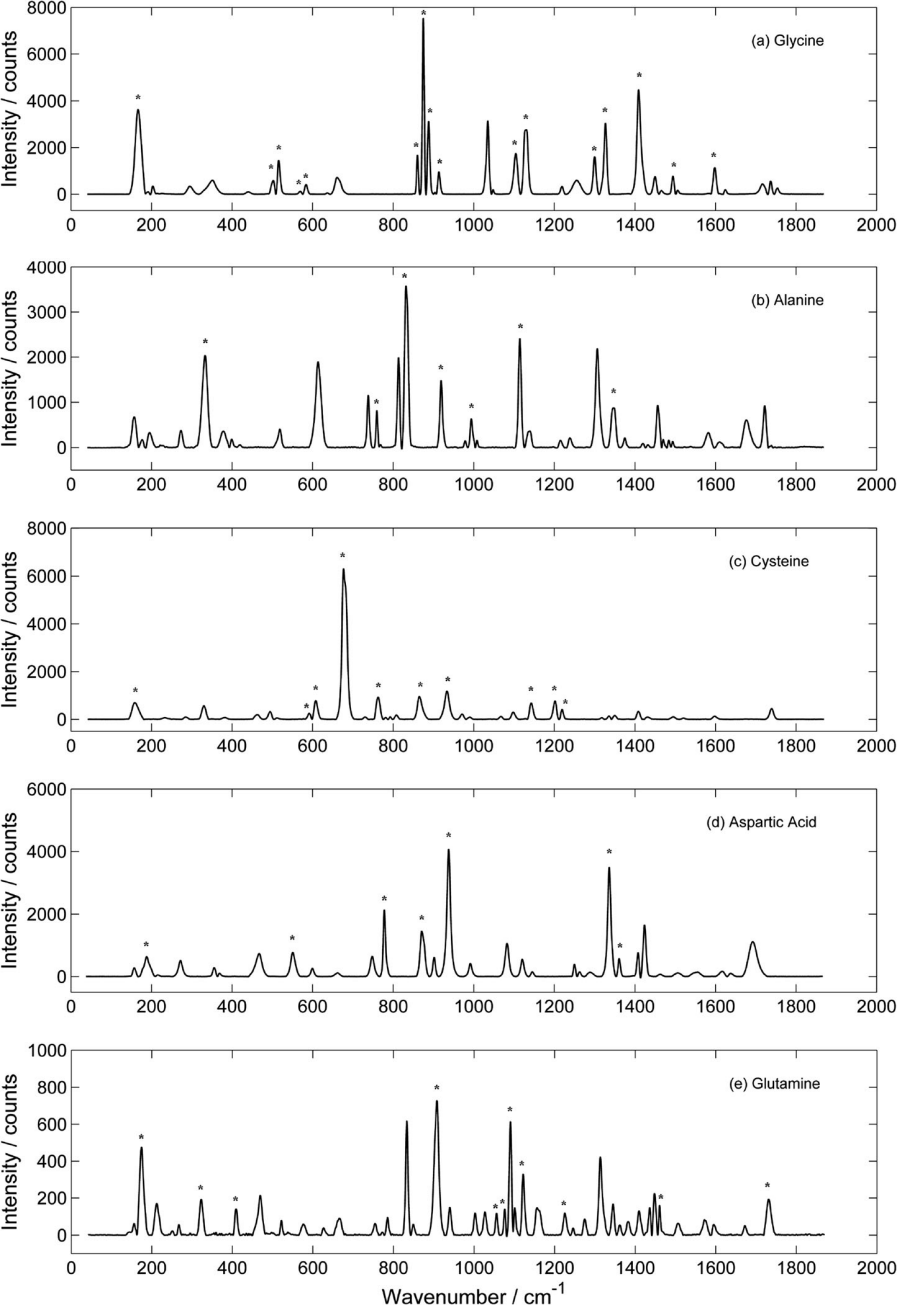
The Raman spectra, averaged over the 16 points taken in map 1 using the 473 nm laser, of the amino acids in this study. The *asterisks* indicate the characteristic bands as described in the Tables 1 and 2

The Raman spectra for alanine, aspartic acid, cysteine, glutamine and glycine are presented in Fig. 1 as references for these amino acids. In the Figure, the characteristic bands, as described in the following sections, are marked with an asterisk. These spectra are an average of a 16 point map using the 473 nm laser, where each point is an average of five 15 s spectra acquisitions as described in Calibration and Data Collection. The baseline of the resulting spectra were corrected for background noise and other factors (described in Calibration and Data Collection) to provide a baseline for band intensity and allowed for more accurate band fitting by the fitting algorithm in *LabSpec5*. The spectral shapes of alanine, aspartic acid, glycine shown in Fig. 1 are in agreement with previously published data (Zhu et al. 2011), as well as that of glutamine (Dhamelincourt and Ramirez 1993). The cysteine spectra presented in Fig. 1 is consistent with that determined by Pawlukojć et al. (2005), except most of the bands recorded in this study are not as intense as the previously published bands.

Detailed statistical results are presented for aspartic acid in Table 1; with the results for alanine, cysteine, glutamine and glycine derived from the same methodology, summarised in Table 2, listing only the characteristic and tentative bands. The rejected bands are not displayed. For ease of description, bands are referred to by a wavenumber position in the text, tables (column: Band name) and figures by taking the average of all the bands across the 16 spectra and rounding to the nearest whole wavenumber; where there were differences in the mean band position between the 514 and 473 nm wavelengths the bands are referred to using the 473 nm nearest whole wavenumber.

**Table 1:**
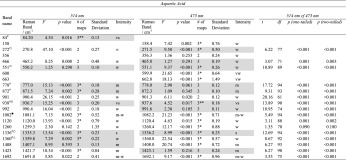
Statistical tests for bands associated with aspartic acid

Those highlighted in grey are the bands that are characteristic or tentative bands of aspartic acid. The rest are rejected as bands for characterising aspartic acid, but could be used as secondary identifying bands. The *F* value, *p* value and the number of maps the band appears in is shown for the two excitation wavelengths, 514 and 473 nm. One- and two-tailed *t*-tests were also performed showing the degrees of freedom (*df*), *t* and *p* values for 514 nm *cf* 473 nm. The bands are significantly different from one another between the two wavelengths, except for the 1260 cm^-1^ band

^#^ Characteristic band assignment within error in the 514 nm spectra

^○^ Characteristic band assignment within error in the 473 nm spectra

^■^ Tentative assignment in 514 nm spectra

^∆^ Tentative assignment in 473 nm spectra

* *p* > 0.05 on one out of three post-hoc Tukey-Kramer tests

** *p* > 0.05 on two out of three post-hoc Tukey-Kramer tests

*** *p* > 0.05 on three out of three post-hoc Tukey-Kramer tests

**Table 2:** Displayed here are the bands designated as characteristic or tentative bands for identifying alanine, cysteine, glutamine and glycine

Alanine																
Band name	514 nm						473 nm						514 nm *cf* 473 nm			
	Raman Band/cm^−1^	*F*	*p* value	# of maps	Standard Deviation	Intensity	Raman Band/cm^−1^	*F*	*p* value	# of maps	Standard Deviation	Intensity	*t*	*df*	*p (one-tailed)*	*p (two-tailed)*
71	71.4	0.47	>0.5	3	0.07	vs										
91	91.1	2.80	0.072	3	0.45	m										
124^a^	124.3	7.97	0.001	3*	0.20	vs										
333^c^	332.1	132.48	<0.001	3*	1.01	m-w	332.9	1.11	0.341	3	0.30	m	4.01	87	<0.001	<0.001
760^d^	759.4	3.31	0.046	3***	0.25	w	759.8	0.00016	>0.5	2	0.00035	m-w	3.16	71	0.001	0.002
832	831.5	5.79	0.006	3*	0.62	vs	832.1	8.30	<0.001	3*	0.53	vs	−4.49	88	<0.001	<0.001
919^a^	918.9	162.75	<0.001	3^e^	0.46	w							−3.52	72	<0.001	<0.001
995^ad^	995.0	5.99	0.005	3**	0.42	w	994.9	2.88	0.102	2	0.33	w	−3.60	73	<0.001	<0.001
1114^a^	1113.7	8.33	<0.001	3**	0.22	m	1114.2	1.55	0.224	3	0.22	m	−4.02	87	<0.001	<0.001
1346							1345.7	3.13	0.055	3	0.91	w	1.57	84	0.06	0.12
Cysteine																
Band name	514 nm						473 nm						514 nm *cf* 473 nm			
	Raman Band/cm^−1^	*F*	*p* value	# of maps	Standard Deviation	Intensity	Raman Band/cm^−1^	*F*	*p* value	# of maps	Standard Deviation	Intensity	*t*	*df*	*p (one-tailed)*	*p (two-tailed)*
68	68.5	1.06	0.358	3	0.47	vw										
131	131.1	0.20	>0.5	3	0.06	w										
159							158.8	2.63	0.083	3	0.52	vw				
593							593.1	2.55	0.092	3	0.70	w				
608^b^	608.5	1.65	0.205	3	0.25	w	608.1	3.29	0.048	3**	0.39	w	0.53	85	0.300	>0.5
679	680.3	2.65	0.084	3	0.55	vs	678.5	5.52	0.007	3**	0.89	vs	4.12	53	<0.001	<0.001
762^a^	792.4	12.61	<0.001	3*	0.47	w							0.34	46	0.368	>0.5
866	866.2	1.84	0.173	3	0.51	w	866.2	1.42	0.253	3	0.34	w	−0.14	87	0.443	>0.5
933	932.3	2.64	0.084	3	0.56	w	933.1	0.45	>0.5	3	0.14	w	−3.08	88	0.001	0.003
1142							1142.4	0.45	>0.5	3	0.21	w	−4.92	88	<0.001	<0.001
1202^d^	1203.0	1.74	0.190	3	0.61	w	1202.2	5.45	0.008	3**	0.71	w	2.37	88	0.010	0.020
1220	1220.4	2.37	0.107	3	0.53	vw	1220.2	6.41	0.004	3*	1.02	vw	0.45	86	0.327	>0.5
Glutamine																
Band name	514 nm						473 nm						514 nm *cf* 473 nm			
	Raman Band/cm^−1^	*F*	*p* value	# of maps	Standard Deviation	Intensity	Raman Band/cm^−1^	*F*	*p* value	# of maps	Standard Deviation	Intensity	*t*	*df*	*p (one-tailed)*	*p (two-tailed)*
96^a^	95.9	13.94	<0.001	3*	0.72	m-s										
112^a^	111.6	5.58	0.007	3*	0.50	vs										
122^a^	121.7	18.22	<0.001	3*	0.60	vs										
174^a^	176.1	3.61	0.037	3**	0.59	m-w	173.5	3.60	0.036	3***	0.88	s				
323^d^							323.2	5.48	0.008	3**	0.75	m-w	1.72	90	0.044	0.089
409^cb^	410.2	120.25	<0.001	3*	0.59	m-w	409.1	4.23	0.021	3**	0.17	m-w	12.84	92	<0.001	<0.001
908^d^							907.5	28.61	<0.001	3*	0.66	s	4.02	94	<0.001	<0.001
1055^ab^	1056.5	129.08	<0.001	3*	0.49	m-w	1055.6	19.98	<0.001	3*	0.37	w	8.97	89	<0.001	<0.001
1076^ab^	1076.7	52.41	<0.001	3*	0.49	w	1075.7	13.85	<0.001	3*	0.25	w	12.05	90	<0.001	<0.001
1090^a^	1091.2	29.07	<0.001	3*	0.53	vs	1090.1	2.19	0.124	3	0.17	vs	10.00	94	<0.001	<0.001
1122^ab^	1122.6	18.25	<0.001	3*	0.38	m-w	1121.7	16.18	<0.001	3*	0.35	m-w	9.05	94	<0.001	<0.001
1225							1225.5	1.87	0.168	3	0.21	w	2.05	82	0.022	0.044
1462^c^	1461.3	71.48	<0.001	3*	0.55	m-w							6.01	53	<0.001	<0.001
1732	1732.2	0.15	>0.5	3	0.07	w							6.72	91	<0.001	<0.001
Glycine																
Band name	514 nm						473 nm						514 nm *cf* 473 nm			
	Raman Band/cm^−1^	*F*	*p* value	# of maps	Standard Deviation	Intensity	Raman Band/cm^−1^	*F*	*p* value	# of maps	Standard Deviation	Intensity	*t*	*df*	*p (one-tailed)*	*p (two-tailed)*
73^a^	72.9	16.89	<0.001	2	0.21	m-s										
137^a^	137.3	17.67	<0.001	2	0.43	m-w										
167^a^	168.4	19.49	<0.001	2	0.59	m	167.1	1.73	0.198	2	0.20	m	8.34	62	<0.001	<0.001
501	502.0	3.93	0.057	2	0.66	w	501.5	6.62	0.015	2	0.69	vw	1.54	62	0.065	0.129
516^a^	516.2	39.88	<0.001	2	0.93	w	516.1	4.11	0.052	2	0.20	w	0.42	61	0.339	>0.5
569^a^	569.0	13.63	<0.001	2	0.46	vw	568.9	3.57	0.069	2	0.27	vw	0.16	59	0.436	>0.5
584^ab^	584.2	13.83	<0.001	2	0.35	vw	584.3	54.09	<0.001	2	0.84	vw	−0.86	61	0.196	0.392
860	859.7	3.74	0.065	2	0.10	w	860.2	0.04	>0.5	2	0.01	m-w	5.20	53	<0.001	<0.001
875	874.5	0.61	0.442	2	0.03	vs	874.6	2.45	0.128	2	0.10	vs	5.41	62	<0.001	<0.001
888^ab^	887.4	69.95	<0.001	2	0.42	m	888.0	8.22	0.008	2	0.22	m	5.44	62	<0.001	<0.001
914							913.8	0.00032	>0.5	2	0.0021	w	−5.32	46	<0.001	<0.001
1104^b^							1104.2	4.35	0.046	2	0.24	w	−1.50	46	0.071	0.141
1130^ab^	1129.2	24.62	<0.001	2	0.96	m-w	1129.9	29.30	<0.001	2	0.63	m	−3.24	62	<0.001	0.002
1300^a^	1299.7	7.47	0.010	2	0.11	w	1299.9	0.64	0.430	2	0.06	m-w	−3.17	62	0.001	0.002
1327^a^	1326.8	43.48	<0.001	2	0.35	m	1327.0	0.23	>0.5	2	0.03	m	−1.95	62	0.028	0.056
1411							1410.4	1.60	0.216	2	0.32	m	−0.22	62	0.413	>0.5
1450^a^	1449.8	38.85	<0.001	2	1.16	vw	1449.7	0.21	>0.5	2	0.08	vw	0.62	62	0.268	>0.5
1495							1494.7	0.45	>0.5	2	0.16	w	−2.31	46	0.013	0.026
1598	1598.2	1.02	0.320	2	0.23	w	1598.1	0.65	0.425	2	0.18	w	0.56	60	0.289	>0.5

Secondary identifying bands or rejected bands are not given

^a^ Characteristic assignment within error in the 514 nm spectra

^b^ Characteristic assignment within error in the 473 nm spectra

^c^ Tentative assignment as a characteristic Band in 514 nm spectra

^d^ Tentative assignment as a characteristic Band in 473 nm spectra

^e^ Did not pass anyTukey-Kramer tests

* *p* > 0.05 on one out of three post-hoc Tukey-Kramer tests

** *p* > 0.05 on two out of three post-hoc Tukey-Kramer tests

*** *p* > 0.05 on three out of three post-hoc Tukey-Kramer tests

### The Characteristic Bands of Aspartic Acid

To show the statistical process of eliminating bands from an amino acid species as non-characteristic, a complete list of bands recorded in the spectra of aspartic acid, with full details of the statistical output from the tests on the bands, are given in Table 1. The bands highlighted in grey met the definition of a characteristic or tentative band as defined in The Definition of a Characteristic Band and Statistical Methods. The bands defined as characteristic for the 473 nm excitation wavelength are at 272, 466, 551, 778, 872, 938, 992, 1336 and 1423 cm^-1^. There is a tentative band at 1360 cm^-1^. The characteristic bands for the 514 nm excitation wavelength are at 84, 551, 778, 872, 938, 1336, 1360 and 1408 cm^-1^ and there is one tentative band at 1082 cm^-1^. The bands at 84 and 272 cm^−1^ are outside the “fingerprint” region. The characteristic bands that passed the ANOVA test had a *p* value range of 0.061 to 0.345. The other bands displayed in Table 1, at 158, 356, 600, 663, 901, 1120, 1260, and 1692 cm^-1^, are rejected at both excitation wavelengths for use as identifying bands for aspartic acid as they do not meet the criteria set out in The Definition of a Characteristic Band and Statistical Methods. Three of these bands, at 356, 901 and 1260 cm^-1^, were rejected on the grounds that the bands only appeared in two of the three maps.

### The Characteristic Bands of Alanine

The characteristic bands in the 473 nm excitation wavelength for alanine are 333, 832, 1114 and 1346 cm^-1^, the tentative bands are 760, and 995 cm^-1^. The characteristic bands for the 514 nm laser are 71, 91, 124 (however, these are outside of the “fingerprint” region), 760, 832, 919, 995 and 1114 cm^-1^. A tentative band for the 514 nm excitation wavelength is at 333 cm^-1^. In both excitation wavelengths the strongest band, 832 cm^-1^, did not pass the ANOVA test, but the means of the bands in two of the three maps are within the measurement accuracy and it does subsequently pass the Tukey-Kramer tests. However, the intensity classification of very strong allows for classification as a characteristic band. The *p* value ranges for the characteristic bands in the 514 and 473 nm spectra were 0.072 to >0.5 and 0.055 to >0.5, respectively. Rejected bands for alanine were at 378, 614, 1134, 1213, 1307, 1463, 1375, 1463, 1484, 1667 and 1976 cm^-1^.

### The Characteristic Bands of Cysteine

In the 473 nm excitation wavelength there are eight characteristic bands that can be used as identifiers for cysteine; their positions are 159, 593, 608, 679, 866, 933, 1142 and 1220 cm^-1^. These bands have all been classified as weak or very weak, except the strongest band at 679 cm^-1^. The band at 1202 cm^-1^ is classified as tentative. There were no tentative assignments among the nine characteristic bands defined for cysteine using the 514 nm excitation laser as all but one of the bands passed the ANOVA test; the band at 762 cm^-1^ did not pass the ANOVA test, but the means of the maps were within the measurement accuracy. The strongest band in the 514 nm spectra was at 679 cm^-1^; the other characteristic bands are 68, 131, 608, 762, 866, 933, 1202 and 1220 cm^-1^, these have weak or very weak intensities. Only three of 15 bands recorded were rejected as characteristic bands, at 1336, 1349 and 1407 cm^-1^ (statistical data are not shown), on the basis that they were only in the upper quartile range for two of the three maps.

### The Characteristic Bands of Glutamine

In the spectra recorded with the 473 nm laser, the characteristic bands that can be used to identify glutamine are 174, 409, 1055, 1076, 1090, 1122 and 1225 cm^−1^. A tentative assignment was given to the band at 908 cm^-1^, which has a strong intensity classification. The characteristic bands in the 514 nm spectra are at 96, 112, 122, 174, 1055, 1076, 1090, 1122 and 1732 cm^-1^. Other bands that can be used as tentative identifiers are at 409 and 1462 cm^-1^, which have medium-weak intensity classifications. The 96, 112, 122, 174 and 1732 cm^-1^ bands are outside of the “fingerprint” region of the spectrum. Rejected bands for glutamine are at wavenumber positions: 72, 522, 577, 665, 753, 833, 848, 940, 1003, 1157, 1314, 1361, 1436, 1450 and 1462 cm^-1^ (statistical data are not shown).

### The Characteristic Bands of Glycine

The data for glycine were based on duplicate rather than triplicate samples due to the third sample being inaccessible to the microscope lenses as it dried adjacent to the wall of the Petri dish. Hence, modified criteria for classifying characteristic bands were used as follows: bands were rejected if the standard deviation was greater or equal to 1.00 or if they failed the ANOVA test; otherwise they were accepted as characteristic bands for glycine. The bands that failed the ANOVA test were accepted as characteristic bands if the means of the maps were within the measurement accuracy of ±0.98 cm^-1^. Glycine has the greatest number of characteristic bands of all the amino acid species examined in this study. When examined with the 473 nm excitation wavelength, bands with the wavenumbers 167, 501, 516, 569, 584, 860, 875, 888, 1104, 1130, 1300, 1327, 1411, 1450, 1495 and 1598 cm^-1^ are characteristic bands. With the 514 nm laser, the characteristic bands are 73, 137, 167, 501, 516, 569, 584, 860, 875, 888, 1130, 1300, 1327, 1450 and 1598 cm^-1^. The strongest intensity band is at 875 cm^-1^. Rejected bands that did not meet the criteria for a characteristic band for glycine were at wavenumber positions 192, 351, 663 and 1034 cm^-1^ (statistical data not shown).

### Statistical Comparison of Band Position Due to Excitation Wavelength

One- and two-tailed *t*-tests were performed to compare the consistency in band positions in the spectra taken with the two excitation wavelengths. For alanine, although all but one characteristic band failed the *t*-tests, the mean wavenumber positions for all the bands in each wavelength are within the measurement accuracy of ±0.98 cm^-1^ of each other. The band at 1346 cm^−1^ showed no statistically significant RWD (*t*-test *p* = 0.06, one-tailed; *p* = 0.12, two-tailed); the other bands all had a RWD that were statistically significant (*t*-test *p* < 0.05).

All but one band failed the *t*-test for aspartic acid. The weak band at 1260 cm^-1^ passed both the one- and two-tailed test with *p* values of 0.09 and 0.18, respectively. Nonetheless, the mean positions of the bands at 272, 466, 551, 778, 872, 901, 938, 1082, 1120, 1336, 1360 and 1408 cm^-1^ are all within the measurement accuracy. Those that are not within the measurement accuracy are at 992, 1260, 1423 and 1692 cm^-1^.

For cysteine, four bands (608, 762, 866 and 1220 cm^-1^) showed no statistical significance in the RWD, while four bands (679, 933, 1142 and 1202 cm^−1^) had RWD of statistical significance. All the bands except the bands at 679 and 1142 cm^-1^ were within the measurement accuracy.

For glutamine, the 323 and 1225 cm^−1^ bands showed no statistically significant RWD (*t*-test *p* = 0.089, two-tailed; *p* = 0.022, two-tailed, *p* = 0.044, two-tailed, respectively) between the two excitation wavelengths. The mean wavenumber positions in each excitation wavelength for the bands 409, 1090 and 1732 cm^-1^ are not within the instrument accuracy.

In the *t*-tests, half of the glycine bands had a RWD that was statistically significant (*p* < 0.05); nonetheless all but the band at 167 cm^-1^ are within the measurement accuracy.

## Discussion

Defining multiple characteristic bands for use in identifying amino acids in unknown samples using statistical methods is an aspect of Raman spectral identification that has not been previously addressed. Vandenabeele et al. (2012) alluded to the idea that a minimum number of bands should be defined that would allow for a definite, positive identification of a molecule in an unknown sample (ideally all laboratory observed bands would be observed in a sample, but this would be atypical). However, a minimum number defined by a set fraction or percentage of the total number of bands would be a non-general case (*ibid*). Another possibility could be to insist that all medium-strong or greater intensity bands attributed to a molecule must be observed to make a positive identification. However, here, a set of bands that are statistically characteristic of the molecule, which are independent of the total number of bands or their intensity, have been defined. These laboratory defined sets of multiple characteristic bands are proposed as complementary to current methods of molecule identification in an unknown sample, such as those expected from future planetary missions.

Here, the majority of bands were observed in both sets of spectra, though only some met the definition of a characteristic band in one of the excitation wavelengths. Furthermore, the intra-map intensity of the spectra has been considered and it was noted that the intensity was variable even when recorded at the same excitation wavelength. Given that the excitation wavelength and the concentration of the sample were fixed, the Raman system was calibrated to the same configuration, and the measuring time was constant in this study, it was assumed that background fluorescence contributed to the noise (described in Calibration and Data Collection). The noise in the spectra was minimised during processing using baseline correction, so it is possible that the intensity of the bands were affected by the spectral processing. However, polarisation and sample orientation effects are also known to affect the intensity of the bands. Detailed and mathematical explanations for changes in intensity can be found, for example, in Long 1977 and Ferraro et al. 2003. These details are beyond the scope of this study, but an example of differences in band intensity due to crystal orientation in amino acids was reported by Dhamelincourt and Ramírez (1991), who found for l-glutamic acid that some bands are more or less intense in specific polarisation directions to the extent that some bands appeared to not be present. As crystal orientation or microcrystalline effects were not explicitly taken into account, it is possible that these could have also affected the intensity of the bands recorded in this study.

**Fig. 2:**
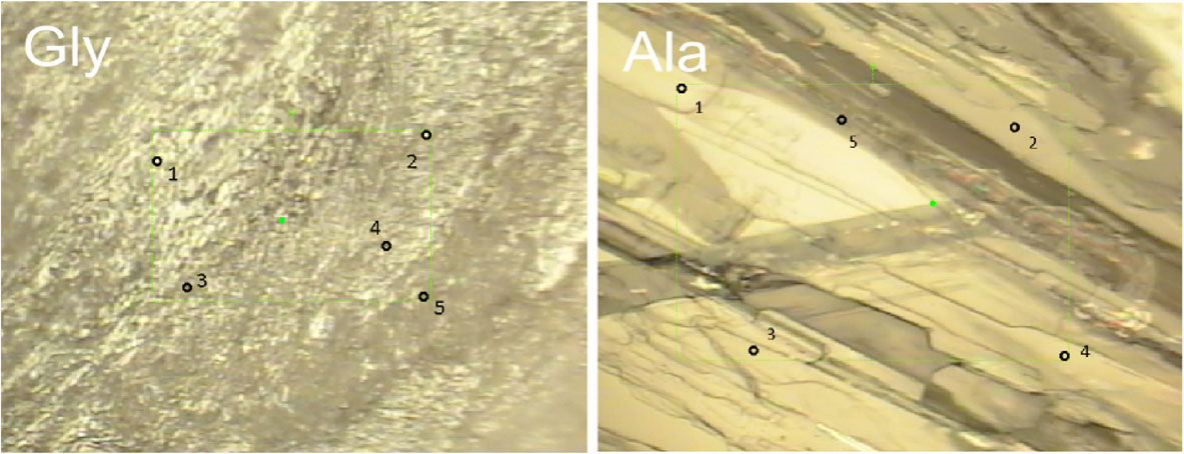
Images of the areas of crystalline glycine and alanine used to conduct a study to determine the effect of orientation on band intensity. This field of view was rotated through 360 ° and spectra were acquired at each numbered point at each angle (0, 90, 180 and 270°)

When designating bands as characteristic for particular amino acids, significant use of the band position was made; however the band intensity was also considered for some tentative assignments (band intensity designations are described in The Definition of a Characteristic Band and Statistical Methods) To investigate the potential contribution of orientation or microcrystalline effects on intensity, a brief study of sample orientation was conducted. The hypothesis was amino acids that crystallise in smaller crystals closer to the size of the laser spot diameter would have a greater variation in intensity compared to those that crystallise in larger crystals when the orientation of the sample is rotated. A sample of alanine (large crystals) and glycine (small crystals) was measured in five positions in a field of view (Fig. 2). The sample was rotated through 360 °, with measurements at the five points taken at 0 °, 90 °, 180 ° and 270 °.

Smaller crystals allow a higher possibility of measuring differing crystal surface effects or the Raman signature over different unit cell orientations, so a wider orientation of bonds would be subject to Raman scattering leading to a higher variety of band intensities. The intensities of the characteristic bands at 90, 180 and 270° (B_1_, B_2_ and B_3_, respectively) were compared to the intensity of the respective characteristic bands at 0° (A) using the relationship:1For alanine, the data for point 3 were poor due to the crystal thinness, no bands were recorded, and only a spectrum representative of the glass substrate was seen. Nonetheless, points 1, 2, 4 and 5 produced good data. The intensities of the bands at points 1, 2 and 5 had intensities with an  value range of 0.40 to 1.80 at all the angles. The 180 ° rotation for point 4 had an  value range of 0.90 to 1.30, demonstrating a 40 % difference in intensities of the bands due to the rotation, similar to the other points; however, for point 4, the 90 ° and 270 ° rotations had intensities approximately 440 and 480 % different, respectively, than the intensities of the bands at 0 °, suggesting that a either a surface effect varying from crystal to crystal or different molecular orientation had been observed at right angles to the 0 ° and 180 ° rotations. For glycine, the  value range for the intensities of the bands at points 1, 2, 3 and 4 is 0.27 to 2.68, which is larger than that seen for alanine. This suggests that the hypothesis that amino acids that crystallise with smaller crystals closer to the size of the laser spot diameter would have a greater range of intensities if rotated than those with larger crystals is correct. The glycine crystal also has an anomalous point; the 0 ° position at point 5 has intensities with an  value range of 0.26 to 5.85. This is a larger range than the alanine anomalous point (point 4), indicating that either there is a greater surface effect at this point or there are different molecule orientations being observed and further suggesting that the hypothesis is correct.

Ergo, though intensities have only been considered here to help define tentative bands and hence do not affect the results of the characteristic band designations; the brief study of sample orientation that was conducted has shown that individual band intensities can vary by almost six times at the same point on a sample depending on the orientation.

### The Statistical Treatment of the Characteristic and Tentative Bands

#### Aspartic Acid

For the 473 nm excitation wavelength the characteristic bands, 272, 551, 938, and 1336 cm^-1^; and for the 514 nm excitation wavelength the characteristic bands 84, 778, 872, 938, 1336 and 1360 cm^-1^ did not originally pass the ANOVA test, but are categorised as characteristic bands due to the mean wavenumber position of the maps being within the measurement accuracy. The statistical testing was rigorous and occasionally ruled out bands that were within in the measurement accuracy, but by expanding the pass criteria for characteristic bands to include these bands a more comprehensive set of characteristic bands was defined. The bands at 938 and 1336 cm^-1^ were assigned as characteristic within the measurement accuracy (i.e.*,* did not pass the ANOVA test), but have intensity classifications of very strong and strong, respectively. These characteristic bands will be the most useful for identifying aspartic acid. The other characteristic bands have intensity classifications of medium or lower; it is possible that lower intensity bands may be lost in the spectrum noise. Nevertheless, if measured, these bands will be important to confirm the presence of aspartic acid in a test sample.

The following bond assignments are given to the bands: skeleton in plane bend (272 cm^-1^), torsion of the NH_3_^+^ group (466 cm^-1^), in plane COOH bend (551 cm^-1^), out of plane OCO^−^ bending (778 cm^-1^), CH_2_ rocking mode (872 cm^-1^), out of plane OH bend (938 cm^-1^), CC stretching mode (992 cm^-1^), in plane CH bending (1336, 1360 cm^-1^), CO stretch and in plane OH bend (1408 cm^-1^) and symmetric CO_2_^−^ stretching (1423 cm^-1^) (Navarrete et al. 1994). The bands that were rejected were at 158, 356, 901, 990, 1082, 1120, 1260, 1408 and 1692 cm^-1^; these bands were rejected on the basis of only passing one Tukey-Kramer test (though often with a *p* value of >0.5) or only appearing in two of the three maps and were often classified with a weak intensity. Therefore, the bands would likely be lost when observed in a bulk host material, but may be used as secondary identifiers.

#### Alanine

The band at 832 cm^-1^ has the strongest intensity classification. However, it does not pass the ANOVA test and is classified as characteristic due to its intensity. A possible explanation for this is the resolution of the grating allows for a band at approximately 813 cm^-1^ to be resolved in some of the maps, but not often enough to be considered for statistical testing. This band may be the cause of the failure of the ANOVA test by the 832 cm^-1^ band; when the lower band is not resolved the 832 cm^-1^ band may incorporate some of the signal causing the wavenumber position to be unstable.

The bond assignment for the characteristic bands were: 333 cm^-1^ is attributed to the CCN bending mode (Rosado et al. 1997). The 1114 and 1346 cm^-1^ are associated with the rocking of the CH_3_ group (*ibid*) and a C−H and NH bend (Kumar et al. 2006), respectively. The assignment for 832 cm^-1^ is either the C−COO stretching mode (Rosado et al. 1997) or CN and C−C stretching modes (Kumar et al. 2006). The tentative bands have bonds associated with the COO wagging mode or CN stretching (Rosado et al. 1997), or HNC and CCH bend modes (760 cm^-1^) (Kumar et al. 2006); and a CNH bend mode (995 cm^-1^) (*ibid*).

The rejected bands for the 514 and 473 nm spectra were at; 378, 614, 919, 1134, 1213, 1307, 1375, 1463, 1484 and 1677 cm^-1^ and 614, 1134, 1307, 1463, and 1976 cm^-1^, respectively (statistical data are not shown). These bands did not meet the criteria of a characteristic or tentative band; for example, the band at 614 cm^-1^ had a *p* value of >0.5, but only appeared in the upper quartile range in two of the three maps and hence was rejected. Furthermore, these bands have an intensity classification of medium-weak or lower (except the band at 1307 cm^-1^, which is a medium strength band), so would potentially be indistinguishable from background noise within a matrix of material.

#### Cysteine

Cysteine, in comparison to the other amino acids in this study, has the most statistically consistent band positions, with only characteristic bands being assigned in the 514 nm spectra and eight of nine bands being assigned as characteristic with one tentative assignment in the 473 nm spectra. However, all the bands except the strongest band at 679 cm^-1^ have intensity classifications of weak or very weak. This could mean cysteine could be difficult to convincingly identify in a test sample with only one strong characteristic band, where the other characteristic bands are likely to be lost in the spectrum noise.

The bond assignments for the characteristic bands are: the 68 and 159 cm^-1^ bands are assigned to a CC or CO_2_ torsion (calculated) and SH torsion, respectively (Pawlukojć et al. 2005). The 593 cm^−1^ band is computed as the CC stretch, but is unobserved empirically by Pawlukojć et al. (2005); the most intense band at 679 cm^-1^ is assigned to the CS stretch; the 866 and 933 cm^−1^ bands are assigned to the CC stretch and SH bending mode, respectively; 1142 cm^−1^ is possibly associated with the CH bend; and the 1203 cm^−1^ band is allocated to the CH_2_ twist (*ibid*).

#### Glutamine

The 514 nm characteristic band positioned just outside the upper limit of the “fingerprint” region (500 to 1700 cm^-1^) at 1732 cm^-1^ has a tentative bond assignment of the C=O stretch (Dhamelincourt and Ramirez 1993). The bands that lie outside the lower limit of the “fingerprint” region at 96, 112, 122 and 174 cm^-1^ have bonds that are associated with CH_3_−CH_2_ and CH_2_−CH_2_ torsion and CCC deformation modes (Dollish et al. 1974). The bonds within the “fingerprint” region at 1055, 1076, 1090 and 1122 cm^-1^ are attributed to the CC stretch (1055 cm^-1^), CN stretch (1076 and 1090 cm^-1^) and the NH_3_^+^ bend and rock modes (1122 cm^-1^) (Dhamelincourt and Ramirez 1993). The tentative band at 1462 cm^-1^ has a band assignment of the CH_2_ bend and scissor modes (*ibid*). The band at 1090 cm^-1^ is has the strongest intensity within the “fingerprint” region and hence will be the easiest band to identify in the spectra of a test sample. Although it was not statistically accepted as a characteristic band, it was within the measurement accuracy so was added to the set of characteristic bands for glutamine. The characteristic bands in the 473 nm spectra at 409 and 1225 cm^-1^ are associated with the skeletal bend, and the CH_2_ bend and twist, respectively (*ibid*). Tentative bands at 323 and 908 cm^-1^ are attributed to the skeletal bend, and a mix of CH_2_ bending and rocking mode, respectively (*ibid*).

#### Glycine

There are more identifying bands attributed to glycine compared to the other molecules in this study. However, these characteristic and tentative bands are statistically less well constrained than the other molecules, due to the statistical tests only being performed on a duplicate sample rather than in triplicate as with the other molecules. Therefore, it is possible that some of the bands that are considered as identifying bands in this duplicate study may have been ruled out if they had been treated in triplicate. Hence, we suggest some caution when attempting to identify glycine using these defined bands. Nonetheless, the bands measured in both excitation wavelengths have a range of intensities from very weak to very strong, which could imply that identification of glycine may be easier than, for example, cysteine, which has only one very strong band and the other characteristic bands’ intensities are classified as weak or very weak.

The bond assignments for the characteristic bands for glycine are: the COO^−^ bend and CH_2_ bend (501 cm^-1^); the COOH bending mode is possibly associated with the 569 cm^-1^ band; the COOH bend is calculated for the 584 cm^-1^ band, but not empirically observed by Kumar et al. (2005); the NH_2_ and CH_2_ twist modes (888 cm^-1^), a CH_2_ deformation (1300 cm^-1^), a mix of the NH_2_ twist and the CH_2_ twist (1327 cm^-1^), the CH_2_ scissor mode (1411 cm^-1^); the CH_2_ and OH bending modes (1495 cm^-1^); and the 1598 cm^-1^ band is assigned to the NH_2_ scissor mode (*ibid*).

### Statistical Comparison of Band Position and the Relative Wavenumber Difference Due to Excitation Wavelength

**Fig. 3:**
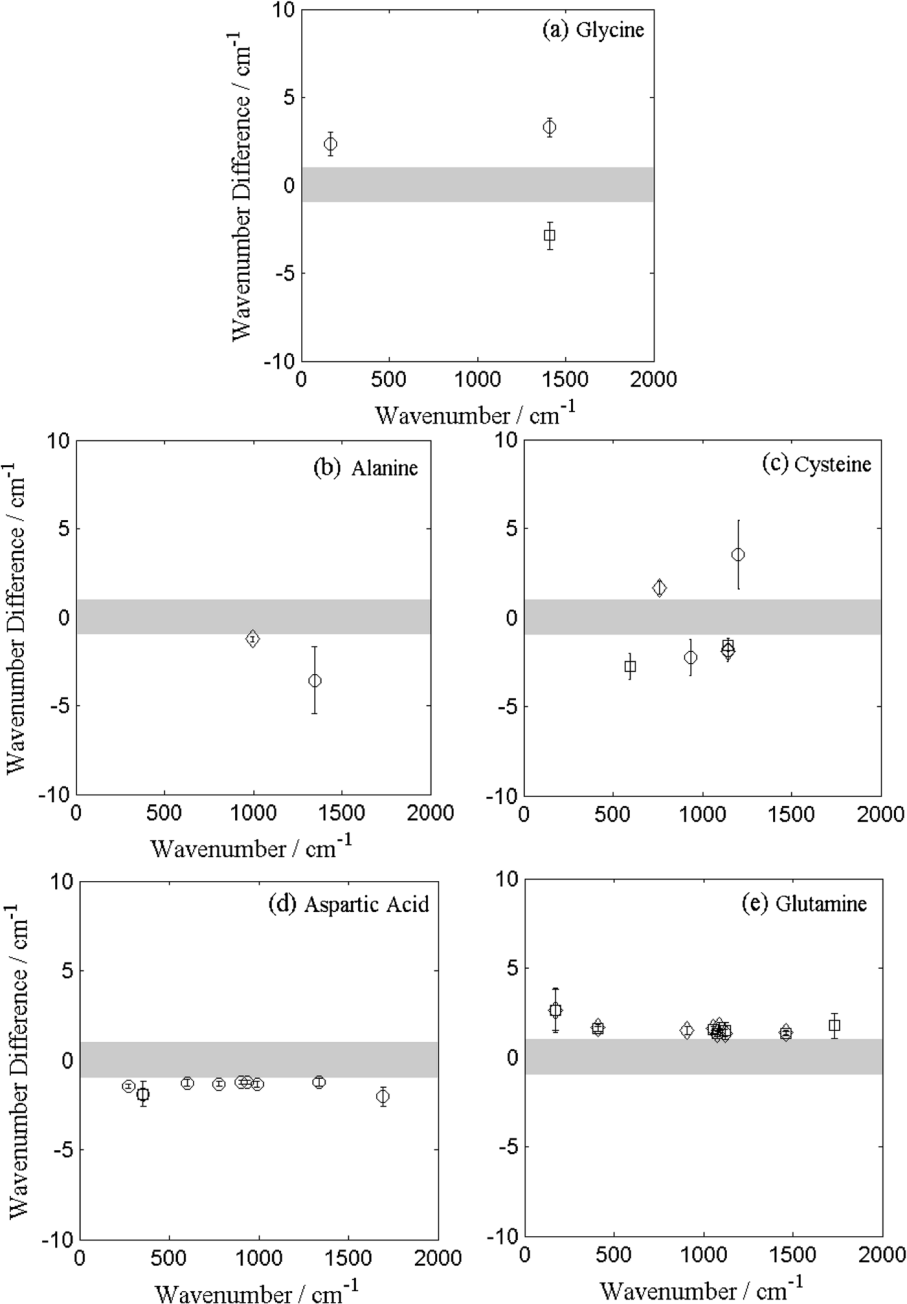
The relative wavenumber difference (RWD) in the position of the characteristic bands in the 514 nm excitation wavelength spectra relative to the 473 nm excitation wavelength spectra for (**a**) glycine; (**b**) l-alanine; (**c**) l-cysteine; (**d**) l-aspartic acid; (**e**) l-glutamine. The *grey area* represents the spectral resolution of the instrument (measurement accuracy). The *error bars* show the standard deviation of the bands from measurements of the samples in the 514 nm laser. *Open circles* represent band positions from map 1; *open squares*, map 2; and *open diamonds*, map 3. The RWD of some amino acids showed a trend to higher relative wavenumbers, some a trend to lower relative wavenumbers and others experience no observable trend. Data for bands within the measurement accuracy and rejected bands are not shown

In this study the consistency of the band position influenced the designation of characteristic bands, so *t*-tests were performed to understand to what extent the excitation wavelength contributed to the wavenumber position of the bands. It was evident that there were some statistically significant differences in band positions for each of the samples studied. This is illustrated in Fig. 3, which shows the difference in band positions of the 514 nm compared to the 473 nm laser spectra. In Fig. 3 the intra-wavelength band positions were also examined, with the error bars representing the standard deviation in the wavenumber position of the band in the 473 nm triplicate map spectra.

The relative wavenumber difference (RWD) for glycine, alanine and cysteine did not show a trend; the data generally lay within the instrument spectral resolution, with few outliers; hence the difference in excitation wavelength had no effect on the consistency of the Raman band positions of these molecules. In comparison to the other molecules, the RWDs of aspartic acid showed a trend towards lower wavenumbers and glutamine showed the opposite trend to aspartic acid, towards higher wavenumbers (see Fig. 3).

To understand any inconsistency in the band positions due to excitation wavelength, the RWD differences were scrutinised by examining the results of the *t*-tests. The characteristic bands of glycine that were subjected to the *t*-test failed more often than passed, which suggested that there was an effect due to the excitation wavelength; however, when the average of the means were compared, all but one band (167 cm^-1^) were within the measurement accuracy, highlighting further that the Raman bands were not affected by the excitation wavelength. The mean positions of the band at 167 cm^-1^ were outside the measurement accuracy by 0.36 cm^-1^, which for many Raman spectrometers is insignificant; in this respect the band can still be considered as characteristic of glycine. Similarly, for the characteristic bands of alanine, all but one band originally failed the null hypothesis. Nonetheless, when means of the band positions in each excitation wavelength were compared they all were within the measurement accuracy; hence supporting that they were not affected by the excitation wavelength.

Half the characteristic bands for cysteine failed the *t*-tests, however all but two had their comparative means within the measurement accuracy. The two bands outside the measurement accuracy were at 679 and 1142 cm^-1^, with differences in their wavenumber position between excitation wavelength spectra of 1.88 and 1.04 cm^-1^, respectively. These wavenumber differences are significant with Raman spectrometers with high resolution gratings, such as the one used in this study, but it is possible that lower resolution gratings, such as those that are likely to be used on future planetary missions, would not measure such a difference and the bands would remain classified as characteristic.

All the characteristic bands of aspartic acid failed the *t*-tests; the results demonstrated that the band positions were statistically significantly different from one another when compared across the two excitation wavelengths and supports the trend to lower wavenumbers seen in Fig. 3. However, when the measurement accuracy is considered, most bands had mean wavenumber positions within the measurement accuracy, except those at 992, 1260, 1423 and 1692 cm^-1^. These bands were outside of the measurement accuracy margin by 0.16, 0.11, 0.37 and 0.12 cm^-1^, which is negligible for lower resolution Raman spectrometers, hence these bands should still be considered as characteristic of aspartic acid.

For glutamine, the *p* values that resulted from the *t*-tests showed that the difference in the band positions between excitation wavelengths is statistically significant for the majority of bands. Nonetheless, all but three of the bands were within the measurement accuracy; the bands 409, 1090 and 1732 cm^-1^ were outside the error by 1.15, 1.08 and 1.16 cm^-1^. For lower resolution Raman spectrometers, is not significant enough to remove these bands from the set of characteristic bands for glutamine, but should be considered with caution by higher resolution Raman spectrometer users. The recorded band positions in each excitation wavelength are listed in Tables 1 and 2.

When considering future planetary missions that have excitation lasers extensively outside of the range 514 to 473 nm (i.e.*,* in red optical, IR or UV wavelengths), the same statistical tests should be applied to laboratory measured Raman bands of target biomarkers to ensure the which bands can be defined as characteristic as they may differ to the ones reported here.

Dispersion effects due to the crystal structure may account for differences in band position. This effect has been shown to occur in disordered carbons and graphites (Vidano et al. 1981; Matthews et al. 1999), where the positions of some of the bands are dependent on the excitation wavelength whereas other band positions are not. In carbon structures, the RWD for the D band is relatively small (about 35 cm^-1^) when examined over a wavelength range between 488 and 647.1 nm (Vidano et al. 1981). This effect, though small, could explain the RWD between the two excitation wavelengths in this study. Matthews et al. (1999) present a model that describes how the dispersion of the D band is due to a resonant Raman process in which phonons and electrons experience a coupling within the Brillouin zone and are in resonance with the excitation wavelength. The C−C bonds and/or the increasing molecular size of the amino acids (i.e.*,* increasing the number of phonons that could resonate with the exCitation wavelength) could create a similar behaviour in the crystalline structures studied here. As the number of C−C bonds (glycine, two; alanine and cysteine, three; aspartic acid, four; and glutamine, five) and number of overall bonds increase, potentially allowing for the dispersion effect to occur more readily, increased scattering and trends in the difference of the wavenumber positions due to excitation wavelength to higher or lower wavenumbers are seen (Fig. 3). For this resonance in the Brillouin zone with the excitation wavelength to be possible in amino acids, the group velocity of the acoustic phonons in the molecules and ratio of the difference in wavenumber position verses the difference in the laser wavelength would have to be similar, as discussed in the model for the dispersion effect in the D band by Matthews et al. (1999). These calculations are beyond the scope of this study, but a dispersion effect of this kind in amino acids crystals has not been previously reported.

## Conclusions

In this study we have emphasised the need to define a set of identifying characteristic bands for astrobiologically relevant molecules, such as the proteinogenic amino acids. In previously published work analysis of Raman spectra is often qualitative and conducted at one excitation wavelength across a number of different Raman spectrometer systems. This leads to discrepancies in the reporting of band positions, which, in an unknown sample may lead to an incorrect or non-identification of a particular molecule, especially as often only the strongest band is acknowledged as an identifier of the molecule.

In this study the astrobiologically relevant amino acids l-alanine, l-aspartic acid, l-cysteine, l-glutamine and glycine were examined. We have quantitatively characterised several bands per molecule that could be used to positively identify the amino acids (Tables 1 and 2) in a future unknown sample. Comparing the 514 and 473 nm band positions did show a statistically significant difference in the relative wavenumber differences (RWDs) for some of the bands in the spectra – one to higher relative wavenumbers (glutamine) and one to lower relative wavenumbers (aspartic acid); the band positions of three of the molecules were consistent within the measurement accuracy of the spectrometer (glycine, alanine and cysteine), however an increase in the scattering of the data was observed, respectively. Nonetheless, the differences were small enough (<2 cm^-1^) that studies using lower resolution Raman spectrometers would be able to use the defined characteristic bands. Any future missions using excitation wavelengths in the red optical, IR or UV should repeat these statistical tests to confirm the characteristic bands at these higher/lower excitation wavelengths. The Raman activity of bonds in a molecule can differ between excitation wavelengths, so the characteristic bands could be different outside of the excitation wavelengths considered in this study.

Another factor that could affect the identification of a molecule in an unknown sample is the orientation or the angle of the crystal, which affects the intensity of the bands. This study has shown that the intensity of bands can differ by up to six times due to the orientation of the sample. Bands that could be characteristic of a molecule but had a reoccurring low intensity were not likely to be treated statistically, due to criteria discussed in Section 2.3. To reduce this effect, future studies defining characteristic bands for astrobiologically relevant molecules should take crystal orientation into account by rotating the sample or recording spectra at several points in the sample. The crushing system on board the ExoMars will powder samples before Raman analysis is performed, with the grain sizes of approximately 20 to 200 μm (Edwards et al. 2012a). The diameter of the spot size will be 50 μm (*ibid*), so the larger grains could potentially encounter issues with orientation effects of the minerals and possible biomarkers that may be contained in the sample.

Amino acids are a target in the search for life elsewhere in the Solar System and Raman spectroscopy could detect molecules pertaining to evidence for life. Future planetary surface missions that intend to use Raman spectroscopy, including that on board the ESA ExoMars rover due for launch in 2018 and a NASA rover mission scheduled for 2020, may use differing excitation wavelengths. The grating used in this study has a high resolution in comparison to those likely to be used on future planetary missions. Hence, these results are providing an upper limit to the characteristic bands for use in identifying these amino acids in unknown samples, without having to define a minimal number of bands required to provide a positive identification of a molecule. The identification of substances relies on knowing accurately and unambiguously the band positions of potential target molecules in those spectra. Therefore, as an aid for identifying target molecules, defining the multiple characteristic bands using statistical methods of a molecule is crucial, otherwise an incorrect identification or a non-identification could occur of the minerals or organic molecules that are important in the search for evidence of life elsewhere in the Solar System.
